# Protective Role of Leaf Variegation in *Pittosporum tobira* under Low Temperature: Insights into the Physio-Biochemical and Molecular Mechanisms

**DOI:** 10.3390/ijms20194857

**Published:** 2019-09-30

**Authors:** Zhilu Zhang, Zhonghua Liu, Haina Song, Minghui Chen, Shiping Cheng

**Affiliations:** College of Chemistry and Environmental Engineering, Pingdingshan University, Pingdingshan 467000, China; hphy226@sohu.com (Z.L.); , zzl1111@tom.com (M.C.); chengship@163.com (S.C.)

**Keywords:** cold response, *Pittosporum tobira*, leaf variegation, linoleic acid, ROS scavenging enzyme, heat shock protein

## Abstract

Leaf variegation has been demonstrated to have adaptive functions such as cold tolerance. *Pittosporum tobira* is an ornamental plant with natural leaf variegated cultivars grown in temperate regions. Herein, we investigated the role of leaf variegation in low temperature responses by comparing variegated “Variegatum” and non-variegated “Green Pittosporum” cultivars. We found that leaf variegation is associated with impaired chloroplast development in the yellow sector, reduced chlorophyll content, strong accumulation of carotenoids and high levels of ROS. However, the photosynthetic efficiency was not obviously impaired in the variegated leaves. Also, leaf variegation plays low temperature protective function since “Variegatum” displayed strong and efficient ROS-scavenging enzymatic systems to buffer cold (10 °C)-induced damages. Transcriptome analysis under cold conditions revealed 309 differentially expressed genes between both cultivars. Distinctly, the strong cold response observed in “Variegatum” was essentially attributed to the up-regulation of *HSP70/90* genes involved in cellular homeostasis; up-regulation of *POD* genes responsible for cell detoxification and up-regulation of *FAD2* genes and subsequent down-regulation of *GDSL* genes leading to high accumulation of polyunsaturated fatty acids for cell membrane fluidity. Overall, our results indicated that leaf variegation is associated with changes in physiological, biochemical and molecular components playing low temperature protective function in *P. tobira*.

## 1. Introduction

Leaf variegation has been observed in many species of higher plants [1,2,3] and this special attractive trait has become a focus of plant breeding as it increases the economic value of ornamental plants [4]. There are two categories of leaf variegation in plants: structural-related variegation and pigment-related variegation [5,6]. Two different types of structural variegation have been described, including the air-space type and epidermis type of variegation, which play adaptive roles to varying light conditions [7]. Pigment-leaf variegation is most common in ornamental plants because of the chlorophyll-deficiency [6]. It is marked by the existence of sections that contain abnormal plastids [5]. The leaf color variegation in plants are divided into several types based on color classification for instance green, yellow and albino (white) sectors on leaves [1,2,8,9]. 

Nuclear and plastid mutations or changes in expression of several genes which contribute to chloroplast biogenesis and chlorophyll biosynthesis induce the leaf variegation [10,11]. The white sectors of variegated leaves lack photosynthetic activity, therefore, leaf variegation may affect photosynthetic efficiency [12]. Previously, a transcriptome study of the *Arabidopsis* white-green variegated mutant *immutans* (*im*) and an *Arabidopsis FtsH2* mutant line (*var2*) revealed that the genes related to photosynthesis were down-regulated in the white sectors of leaves [13,14]. Furthermore, the chlorophyll-deficient leaf-mutant also showed the expressional repression of transcriptional factors *GLK1*, *Ftsz* and *MinD* that regulate chloroplast development and division [15]. Recently, a mutation in the transcription factor mitochondrial transcription termination factor (*mTERF*) has been found to induce colorlessness in leaves of variegated fig [3]. Although these studies have provided a deep understanding of the variegation mechanism in plants, the advantage of this trait for the good fitness or for the plant physiology is still poorly understood.

Several potential physiological advantages of variegation have been proposed in plants. For example, it was reported that leaf variegation is involved in plant defense from enemies including aposematic coloration, mimicry of dead or infested plants, masquerade and camouflage [16,17,18,19]. It can also play physiological roles such as improved water or gas transport [20], mitigation of UV radiation [21] and thermoregulation [22]. Investigations led on forest trees displaying variegated leaves hinted that the trait might be a strategy to prevent the attack of herbivores [23,24]. Later, studies by Mwafongo et al. [25] on leaf variegation patterns in *Ledebouria revoluta* highlighted two possible functions including the photoprotection role and the aposematic role. Very recently, Shelef et al. [22] demonstrated that under lower temperatures, variegated wild type *Silybum marianum* leaves were significantly warmer than all-green mutants, conferring cold stress tolerance. These studies showed that variegation in plants is not just a color mutation but has some physiological advantages.

*Pittosporum tobira* (Thunb.) Aiton belonging to the family Pittosporaceae originated from East Asia and at present is being widely cultivated as an ornamental flowering plant in temperate and subtropical regions around the world [26]. Typically, *P. tobira* plants are about 2–3 m high with thick, rubbery and dark green colored leaves. The fragrant flowers of *P. tobira* have been well studied for their antimicrobial and anti-oxidant activities [27,28]. Importantly, some cultivars exhibit leaf variegation with yellowish or creamy white leaf margins and green interior, which have a greater aesthetic appeal and ornamental value compared to the typical all-green *P. tobira*. These particular variegated cultivars are spread to temperate regions. However, besides the aesthetic advantage, the intrinsic physiological importance of leaf variegation for *P. tobira* is unknown.

In the present work, we studied two *P. tobira* cultivars namely, “Variegatum” and “Green Pittosporum” with distinct leaf coloration features. To thoroughly understand the role of leaf variegation in *P. tobira* under cold condition, we investigated the physio-biochemical characteristics at different temperature gradients and profiled leaf transcriptome of the two cultivars under cold stress. Our findings elucidate the leaf variegation mechanism in *P. tobira* and provide novel insights into the thermo-protective function of this important trait.

## 2. Results

### 2.1. Characteristics of Variegated Leaves in Pittosporum Tobira

**Figure 1:**
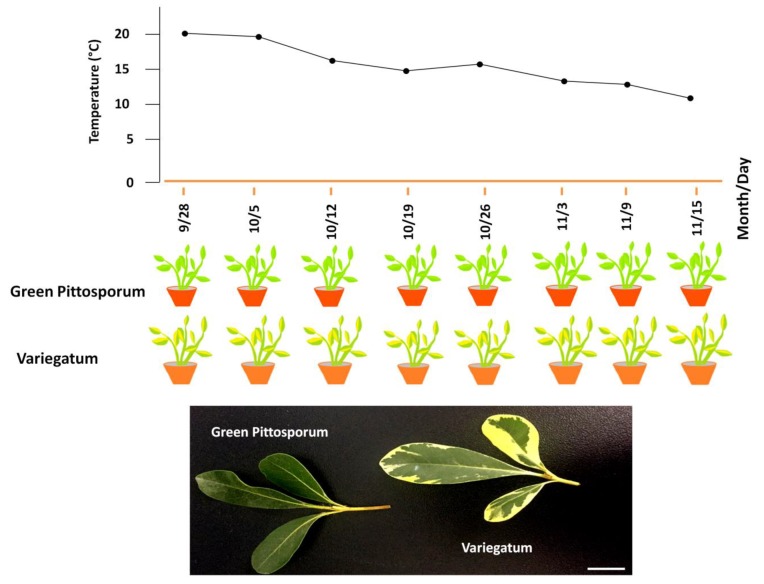
Overview of the experiment design and phenotypes of the two *Pittosporum tobira* cultivars, namely “Variegatum” with green/yellowish variegated leaf and “Green Pittisporum” with complete dark green leaf. Leaf samples were harvested at different dates following decrease of ambient temperature. The bar = 2 cm.

**Figure 2:**
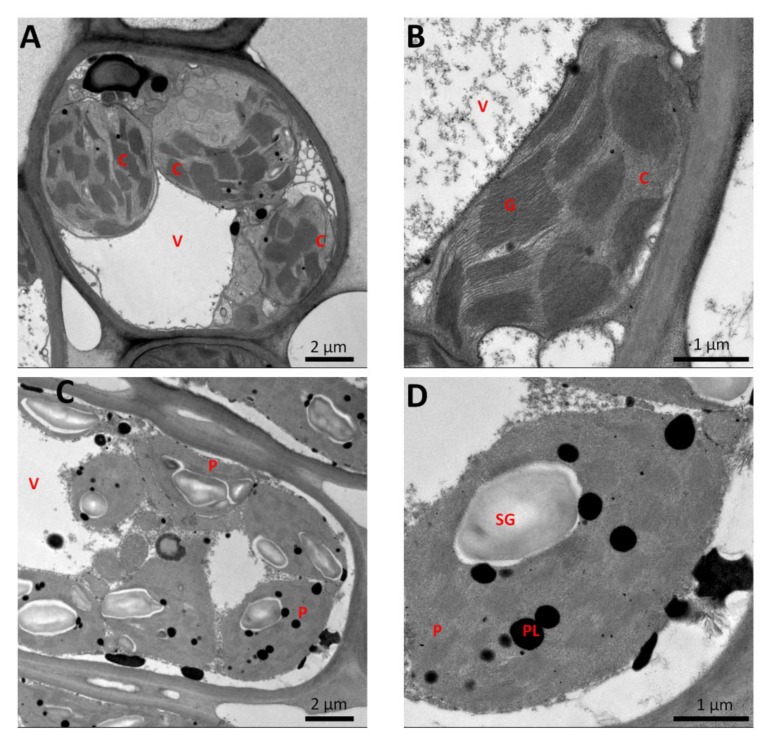
Chloroplast ultrastructure of the green (**A**,**B**) and yellow (**C**,**D**) sectors in variegated leaves of *Pittosporum tobira* cultivar “Variegatum”. C = chloroplast; P = plastid; SG = starch granule; G = grana; V = vacuole, PL = plastoglobuli.

**Figure 3:**
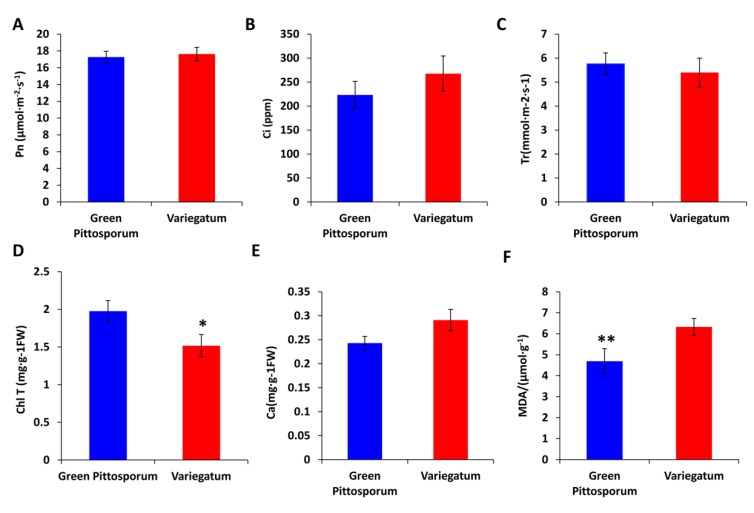
Physio-biochemical comparison of leaf from variegated “Variegatum” and non-variegated “Green Pittosporum” cultivars. (**A**) net photosynthetic rate (*Pn*), (**B**) intercellular CO_2_ concentration (*Ci*), (**C**) transpiration rate (*Tr*), (**D**) total chlorophyll content (ChlT), (**E**) carotenoids content (Ca) and (**F**) malonaldehyde content (MDA). *, ** above the bars represent significant difference between the two cultivars at *p* < 0.05 and *p* < 0.001, respectively, using Tukey’s honestly significant difference (HSD) test.

A naturally occurring leaf variegated cultivar of *Pittosporum tobira* named “Variegatum” was collected from Pingdingshan, Henan province in China. The cultivar “Variegatum” bears yellowish margins and green interior leaves, whereas, the typical cultivar “Green Pittosporum” exhibits dark green colored leaves (Figure 1). The phenotypic characteristics such as leaf thickness and shape were found to be similar for both cultivars except for the variegation. It is well documented that the leaves of variegated plants having green/yellow sectors have impaired chloroplast biogenesis, less photosynthetic pigments in the yellow sectors and also accumulate excessive levels of reactive oxygen species (ROS) [29,30]. To verify these observations in *P. tobira*, we analyzed the chloroplast ultrastructure in the yellow sector compared to the green sector of the variegated leaf. As shown in Figure 2A,B, the green sector contained well-developed chloroplasts with stacked grana. In contrast, in the white sector of the leaf, plastids did not contain stacked grana but contained large starch granules and many plastoglobuli (Figure 2C,D). Next, we assessed the photosynthetic parameters and malonaldehyde (MDA) in both cultivars in August when the ambient temperature is around 20 °C (Figure 1). The net photosynthetic rate (*Pn*), the intercellular CO_2_ concentration (*Ci*) and the transpiration (*Tr*) rate were found similar between leaves from both cultivars (Figure 3A–C), showing that the photosynthetic efficiency is not significantly impaired in “Variegatum” as compared to “Green Pittosporum”. Next, we compared the content of photosynthesis-related pigments such as total chlorophyll (chlT) and carotenoids (Ca) in both leaf types. The results revealed that the chlT contents were significantly lower (*p* < 0.05) in “Variegatum” compared to the “Green Pittosporum” (Figure 3D), while Ca was higher in “Variegatum” compared to the “Green Pittosporum” (Figure 3E), indicating that the yellowish phenotype in “Variegatum” is underlined by a reduced chlorophyll content and a stronger accumulation of carotenoids. We further measured the MDA content, which is associated with lipid peroxidation via an increased generation of ROS [31]. The MDA was significantly (*p* < 0.01) and highly accumulated in “Variegatum” leaves compared to “Green Pittosporum” leaves (Figure 3F), implying a high level of ROS in the variegated leaves.

Taken together, our results showed that leaf variegation trait in *P. tobira* is associated with defected chloroplast biogenesis in the yellow sector, reduced chlorophyll content, strong accumulation of carotenoids and high level of ROS.

### 2.2. Effect of Temperature Decrease on ROS-Scavenging Enzyme Activities in P. tobira Cultivars

**Figure 4:**
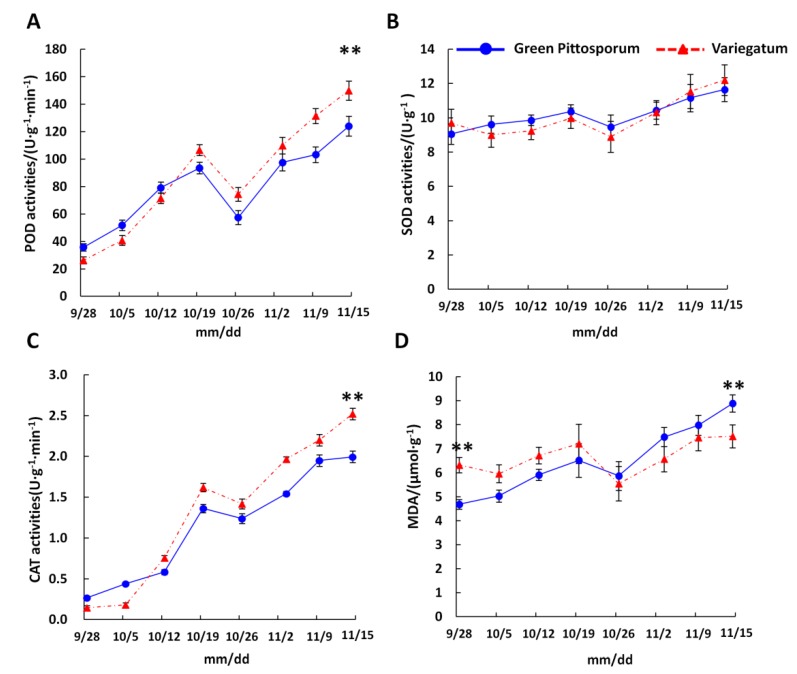
Antioxidant enzymatic activities during temperature decrease. (**A**) Peroxidase activity (POD), (**B**) Superoxide dismutase activity (SOD), (**C**) Catalase activity (CAT), (**D**) malonaldehyde content (MDA). ** above the lines represents significant difference between the two cultivars at *p* < 0.001, using Tukey HSD test.

The natural occurrence of leaf variegation in plants suggests that the trait might have adaptive functions [32]. In line with this, we investigated the enzymatic changes with respect to cold stress response in leaves of “Variegatum” and “Green Pittosporum” cultivars over a period of three months (from August to November) when the ambient temperature decreases from optimal condition (20 °C) to cold condition (10 °C). The results showed that the two cultivars respond similarly to the temperature decrease (Figure 4). The activities of all the three ROS-scavenging enzymes including peroxidase (POD), catalase (CAT), superoxide dismutase (SOD), were increased over the assayed period. Notably, CAT and POD displayed a sharp increase in response to the temperature decrease. At the lowest temperature (10 °C, November 15^th^), POD and CAT activities were significantly higher (*P* < 0.01) in “Variegatum” as compared to “Green Pittosporum” (Figure 4A–C), denoting a stronger response to cold in “Variegatum”. We extended the investigation on the MDA contents in both cultivars in order to record the stress levels induced by the temperature decrease. As expected, MDA levels also increased in both cultivars with the temperature decrease, but “Variegatum” seems to suffer less from cold stress. This is evidenced by the significantly higher (*p* < 0.01) MDA in “Green Pittosporum” when the temperature reached 10 °C (Figure 4D).

Overall, our results indicated that “Variegatum” is endowed with an efficient ROS-scavenging enzymatic system, which is mainly triggered under low temperature. Hence, leaf variegation trait plays a low temperature protective function in *P. tobira*.

### 2.3. Transcriptome Sequencing in Leaves of “Variegatum” and “Green Pittosporum” and Functional Annotation of Unigenes

To get an insight into the molecular pathways and genes conferring the strong response to low temperature in “Variegatum”, we synthesized six cDNA libraries from leaves collected from “Variegatum” and “Green Pittosporum” plants under cold conditions (10 °C) and generated *de novo* RNA-sequencing data for the first time in *P. tobira*.

**Figure 5:**
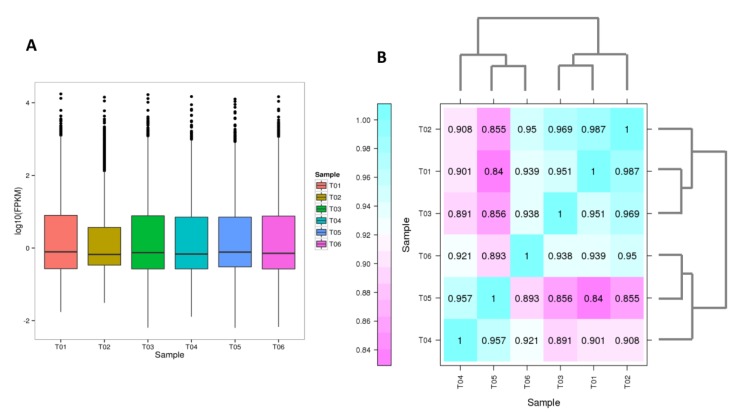
Overview of the transcriptome sequencing. (**A**) Gene expression profile in the 6 libraries. T01-T03 represent the three replicates libraries of the cultivar “Green Pittosporum” and T04-T06 represent the three replicates libraries of the cultivar “Variegatum”. (**B**) Heatmap clustering showing correlation among *P. tobira* different samples based on global expression profiles. Numbers in the heatmap represent the Pearson correlation value.

The RNA-seq yielded a total of 40.88 Gb clean data with 92.78% of bases scoring Q30 and above (Table 1). The assembly was performed using the Trinity software and a total of 112,875 unigenes were obtained with N50 length about 1,017 bp. The assembly integrity was high and specific statistics are shown in Table 2. A total of 51,718 unique genes were functionally annotated based on various databases (Table 3, Table S1). The clean data of each sample was serialized with the assembled unigene libraries and the mapping result statistics are presented in Table 4. Of these genes, 19,677 genes were expressed with the number of fragments per kilobase of exon per million fragments mapped (FPKM) values ranging from 0.04 to 22578.37 (Table S2, Figure 5A).

Hierarchical clustering of the samples based on FPKM showed that all the biological replicates clustered together, suggesting a high reliability of our RNA-sequencing data (Figure 5B). Moreover, a clear separation of the two leaf sample types was observed, implying that a large number of genes may be differentially expressed between the two cultivars to explain the relative stronger response to cold stress in “Variegatum”.

### 2.4. Differential Gene Expression Analysis between “Variegatum” and “Green Pittosporum” under Cold Condition

**Figure 6:**
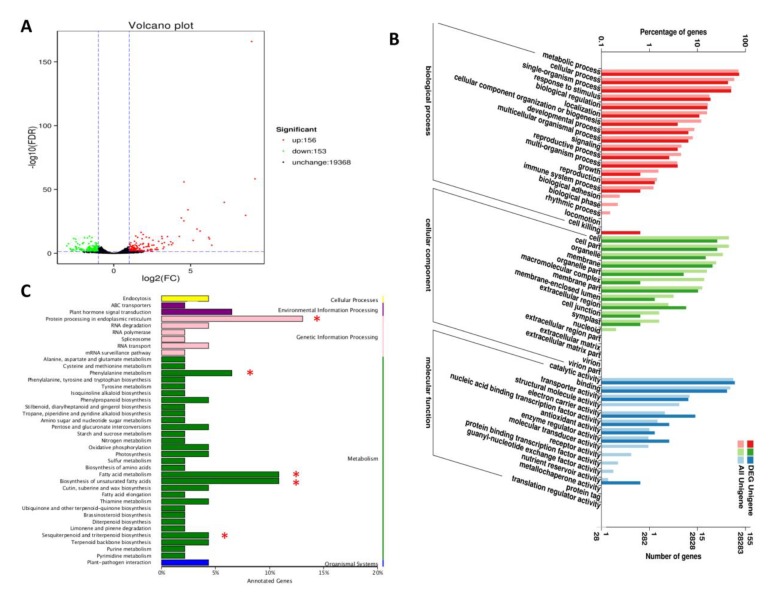
Differentially expressed genes (DEG) analysis between “Green Pittosporum” and “Variegatum”. (**A**) Volcano plot depicting the up-, down- and no- regulated genes between the two cultivars. (**B**) Gene ontology enrichment analysis of the DEGs. (**C**) KEGG enrichment analysis of the DEGs. * represent the significantly enriched pathways.

The differential gene expression analysis was performed on all expressed genes by comparing their expression levels between “Variegatum” and “Green Pittosporum”. As shown in Figure 6A, a total of 309 differentially expressed genes (DEG) were obtained, including 156 up-regulated and 153 down-regulated genes in the variegated leaves. To validate our differential expressed gene result, we selected five up-regulated genes and five down-regulated genes (Table S3) and performed qRT-PCR using the cDNAs from leaves of the two cultivars. The qRT-PCR results were strongly correlated with the RNA-seq data (R^2^ = 0.89, Figure S1). This result confirms well the high reliability of the RNA-seq data obtained in the present study. 

We performed gene ontology (GO) enrichment analysis of these DEGs based on three ontologies: biological process, cellular component and molecular functions. In the biological process components, metabolic and cellular process was found to be the most dominant group (Figure 6B). Within the cellular components, cell and cell part represented the most dominant functional groups. Meanwhile, the catalytic activity and binding were the most abundant functional groups among the molecular functions, showing that enzymes and transcription factors encoding genes may play key roles in the differential cold response. Furthermore, Kyoto Encyclopedia of Genes and Genomes (KEGG) enrichment analysis of the DEGs showed that the biosynthesis of unsaturated fatty acids, sesquiterpenoid and triterpenoid biosynthesis, fatty acid metabolism, phenylalanine metabolism and protein processing in endoplasmic reticulum were the main pathways contributed by the DEGs (Figure 6C). The diversity of these molecular pathways highlights the complex mechanism of the improved cold response in relation with leaf variegation in *P. tobira*.

### 2.5. Major Transcription Factors Differentially Regulated between “Variegatum” and “Green Pittosporum” under Cold Conditions

**Figure 7:**
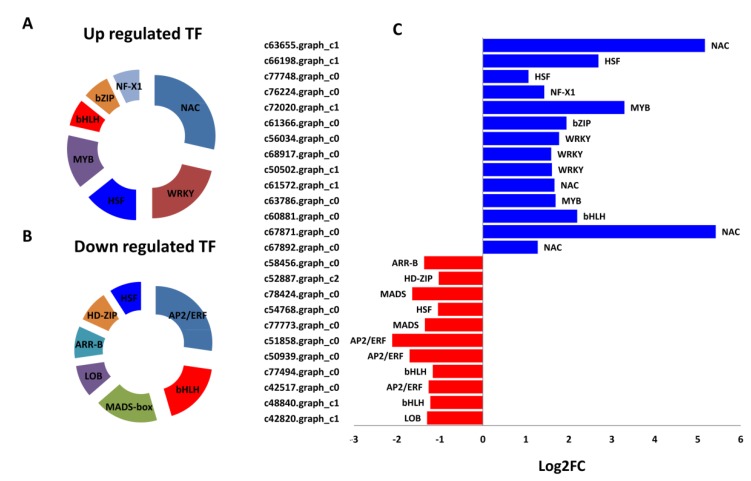
Major transcription factors (TF) families regulating cold response in *P. tobira*. (**A**) up-regulated TFs families in “Variegatum”, (**B**) down-regulated TFs families in “Variegatum”, (**C**) Log2 Fold change of the expression of TF genes.

Since the GO enrichment showed that differential binding activity was important for the cold responses in “Variegatum”, we extended our study over the major transcription families (TF) present within the DEGs. In total, 11 down-regulated and 14 up-regulated TFs in “Variegatum” were detected. Among the down-regulated TFs, AP2-ERF, bHLH and MADS-box TFs were enriched (Figure 7A). Distinct TF families were enriched in the up-regulated genes and included NAC, WRKY, HSF and MYB (Figure 7B). Expression fold change of these TFs showed that two NAC genes (*c67871.graph_c0* and *c63655.graph_c1*) were strikingly up-regulated in “Variegatum” (Figure 7C) and may play prominent positive regulatory roles for cold stress endurance.

### 2.6. DEGs Related to the Biosynthesis of Unsaturated Fatty Acids and Fatty Acids Metabolism 

An increase in polyunsaturated fatty acids has also been reported to play a crucial role in the chilling tolerance of plants [33]. In this study, ten DEGs were mapped to the pathways related to the biosynthesis of unsaturated fatty acids and fatty acids metabolism. Interestingly, all of these DEGs were annotated as endoplasmic reticulum omega-6 fatty acid desaturase (FAD2) and were all up-regulated in “Variegatum” (Table 5). Since the microsomal enzyme FAD2 principally acts on the desaturation of C18:1 to C18:2 [34], we deduce that “Variegatum” strongly accumulates C18:2 in leaf as a protective molecule under cold conditions. Besides, we also detected nine GDSL-Lipase involved in lipid biosynthesis in plants. All the GDSL genes were down regulated in “Variegatum” (Table 5), suggesting a probable opposite function of GDSL and FAD2 genes during cold endurance in “Variegatum”.

### 2.7. Disturbance of Protein Processing in Endoplasmic Reticulum under Cold Conditions

The endoplasmic reticulum is a subcellular compartment where proteins and lipids are folded with the help of chaperones. The enrichment of this pathway (Figure 6C) indicates a disturbance of proteins and lipids synthesis under cold conditions. Nine DEGs, all being heat shock proteins (HSP) were detected within this pathway. Notably, we observed that all the small HSP genes (15–22 kDa) were down-regulated while the high molecular weight HSP genes (70–90 kDa) were up-regulated in the variegated leaves (Table 5). This result highlights the weight dependent roles of HSP genes for a stout cold response in variegated *P. tobira*. 

### 2.8. DEGs in the Phenylalanine Metabolism

In this important pathway, we found three DEGs including two POD genes (*c68309.graph_c0* and *c74970.graph_c1*) and *c55523.graph_c0* annotated as an aminotransferase TAT2. Interestingly, all these genes were up-regulated in “Variegatum”, showing that they contribute positively to the enhanced cold response (Table 5). More importantly, the activation of these genes correlates well with the strong enzymatic activity of POD detected through our biochemical assay in “Variegatum” when the temperature reached 10 °C (Figure 4A).

## 3. Discussion

### 3.1. Characteristics of Leaf Variegation in P. tobira

Leaf variegated plants have green/white (or yellow) sectors and cells in the green sectors contain normal appearing chloroplasts, while cells in the white sectors have impaired chloroplast biogenesis and lack photosynthetic pigments [12]. Moreover, it has been shown that leaf variegated plants accumulate high levels of ROS [29,30]. Although these mechanisms are commonly found in variegated plants, a recent study of the rice *z3* mutant leaves showed a new mechanism of variegation, which was caused by an unbalanced distribution of citrate in a transverse pattern in leaf tissues [34]. In our study, we also noted a defected chloroplast development in the yellow sector, reduced chlorophyll content and a high level of ROS in the variegated cultivar (Figure 2 and Figure 3). We also observed an abundance of starch granules in the yellow sector as compared to the green sector, suggesting that the yellow sectors are nutrient sinks because they are unable to perform photosynthesis. Similar conclusions were previously reported in different species including variegated *Arabidopsis* [1,35,36], tobacco [37], begonia [6] and fig [3]. However, the photosynthetic efficiency was not obviously affected in “Variegatum” (Figure 3), contrasting with the reports that leaf variegation affects photosynthetic efficiency [12]. In fact, the yellowish area on “Variegatum” leaves is located on the margin and has a very low surface coverage. So, an explanation to this observation can be that the green part of the leaf is large enough to ensure the photosynthetic activity. Leaf variegation has been attributed to a deficiency or a significant reduction of photosynthetic pigments including carotenoids. In the *Arabidopsis* white-green variegated mutant *immutans* (*im*), an inhibition of carotenoids formation was observed [38]. Similarly, the white section in leaf of variegated *Epipremnum aureum* contains 10-fold less carotenoids than the green section [39]. In *Cyclamen purpurascens*, the light green leaf stripes were found with reduced carotenoids and chlorophyll contents [40]. In green/yellow patterns variegated species, similar observations were also noticed in *Aucuba japonica* [41] and *Coleus bluemei* [42]. Intriguingly, we observed a higher concentration of carotenoids in the variegated leaves of *P. tobira* as compared to the complete green leaves (Figure 3), a phenomenon which has not yet been reported in variegated plants. Since carotenoids function as accessory light-harvesting pigments, broadening the spectral range over which light can support photosynthesis in plants [43], we deduce that the high carotenoids content in “Variegatum” may compensate the reduced chlorophyll to maintain similar photosynthetic activity as in leaves of “Green Pittosporum”.

### 3.2. Protective Role of Leaf Variegation in P. tobira under Cold Condition

The natural occurrence of variegation in plants suggests that the trait might play some adaptive functions beyond their aesthetic value [32]. It has been suggested that leaf variegation plays several physiological and ecological functions such as defense from enemies, adaptations to light, temperature, etc. [16,17,18,19,20,21,22,23,24,25]. We tested the hypothesis that leaf variegation plays a low temperature protective function in *P. tobira*, which is an ornamental shrub widely grown in temperate climate and therefore is annually subjected to cold stress. It is well known that increased activities of antioxidant enzymes such as POD, CAT, SOD under abiotic stress conditions including drought, salt, chilling, heat, etc., promote enhanced stress tolerance in plants [44]. Our results demonstrated that “Variegatum” has much more efficient ROS-scavenging machinery compared to “Green Pittosporum” and accumulates less MDA, an indicator of limited cellular membrane damage due to lipid peroxidation. Hence, “Variegatum” better tolerates low temperature stress than “Green Pittosporum” (Figure 4). We further sequenced the transcriptomes of both leaf types under cold condition (10 °C). Differential gene expression (DEG) analysis resulted in 309 DEGs between the two cultivars, enriched in biological pathways related to the biosynthesis of unsaturated fatty acids, sesquiterpenoid and triterpenoid biosynthesis, fatty acid metabolism, phenylalanine metabolism and protein processing in endoplasmic reticulum, which may be crucial pathways involved in cold stress alleviation (Figure 6).

Cell membrane structure, integrity and fluidity are affected by lipid composition and the degree of fatty acid (FA) desaturation in plants [45]. It has been documented that changes in unsaturated fatty acids content can improve plant tolerance to environmental stresses such as cold, heat and drought [46,47,48,49,50,51], since modification of membrane fluidity results in an environment suitable for the function of critical integral proteins, such as the photosynthetic machinery, during stresses [52]. In this study, we detected ten FAD2 genes all significantly up-regulated in “Variegatum” leaves under cold condition (Table 5). The microsomal enzyme FAD2 principally acts on the desaturation of C18:1 (monounsaturated FA) to C18:2 (polyunsaturated FA) [53], suggesting that “Variegatum” tends to increase polyunsaturated FA (PUFA) level, a mechanism to maintain cell membrane fluidity under low temperature [54,55]. This skill of adjusting membrane fluidity by varying the unsaturated fatty acid content is characteristic of cold-responsive plants [52]. Cold acclimating potato (*Solanum commersonii*) was found to accumulate linoleic acid (18:2) in the membrane glycerolipids of the leaves, whereas commercial, non-acclimating potato (*Solanum tuberosum*) did not show this trait during cold stress [56]. Our findings are in perfect accordance with reports of Liu et al. [51], who showed that over-expression of tomato FAD2 gene alleviates the photoinhibition of photosystems 2 and 1 and improves tolerance under chilling stress. Similar observations were reported in various plants such as cotton [57], *A. thaliana* [58], *Olea europaea* [59], *Synechocystis* sp. [60], etc., under low temperature conditions. 

Membrane fatty acid composition is, to a great extent, determined by the activities of complexly regulated integral fatty acid desaturases and lipases [52]. GDSL-lipase participates in fatty acid catabolism and studies have shown that the linoleic acid and other PUFAs contents are significantly decreased when GDSL genes are over-expressed [61,62,63,64]. Here, we detected nine GDSL-lipase genes all down-regulated in “Variegatum” under low temperature stress (Table 5), denoting a strategy to keep the high level of PUFA for the maintenance of cell membrane stability. We deduce that down-regulation of GDSL genes and up-regulation of FAD2 genes is therefore an integrated and efficient mechanism to cope with cold stress in *P. tobira* cv. “Variegatum”.

Another group of genes detected within the DEGs between “Variegatum” and “Green Pittosporum” under cold condition are heat shock proteins (HSP) (Table 5). HSPs are molecular chaperones that are constantly present in cells to correctly fold proteins involved in routine cellular processes such as translocation, cell-signaling and metabolism [65]. However, HSPs become abundant in most organisms in response to protein denaturation caused by environmental, metabolic and pathological stresses [66]. For example, *Arabidopsis*, grape, rice, *Brassicas* increase the production of HSPs to augment survival in cold environments [67,68,69,70]. On the other hand, it was reported that a complex coordination of HSPs underlines cold tolerance in plants [65]. In fact, some HSPs are either up- or down-regulated when heat shock factors (HSFs) bind to their promoter regions [71,72]. This suggests that not all HSPs positively participate in cold or stress tolerance in plants. Each group of these HSPs has a unique mechanism [65]. In our study, we observed that small HSPs were all down-regulated while high molecular weight HSP genes were up-regulated in “Variegatum”, pointing out an opposite function of HSPs for cold response in *P. tobira* with respect to their molecular weights. For now, a clear explanation for this phenomenon is yet to be found, hence, an in-depth investigation of the role of HSP genes and their relation with the significantly altered HSF transcription factors under cold condition in *P. tobira* is necessary in order to clarify this intriguing finding.

Our transcriptome analysis also unveiled several peroxidase genes from the phenyalanine pathway as well as some cytochrome *P450* genes from the sesquiterpenoid and triterpenoid biosynthesis as candidate genes, which positively contribute to the enhanced cold responses in “Variegatum” (Table 5). Peroxidase genes have been extensively studied in plants for their ROS-scavenging activity under various biotic and abiotic stresses, including chilling [73,74,75]. Similarly, Liu et al. [76] recently investigated the prominent biological pathways engaged in wild banana tolerance to chilling. They observed significant changes in the sesquiterpenoid and triterpenoid biosynthesis, particularly cytochrome *P450* genes, a finding that supports well the results of our study.

**Figure 8:**
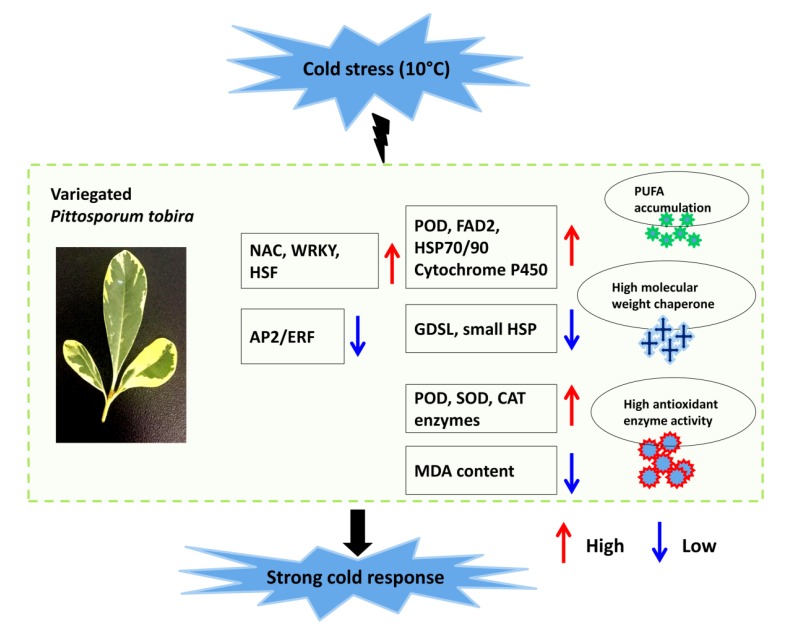
A schematic model of the proposed mechanism underlying the strong cold response in *P. tobira* cv. “Variegatum”.

Taken together, we showed that leaf variegation in *P. tobira* is associated to defected chloroplast development, reduced chlorophyll content, high content of carotenoids and a high level of ROS. The results of transcriptome analysis were consistent with the enzymatic activity under cold conditions. These results pointed out that the leaf variegation trait plays low temperature protective effect in *P. tobira* by inducing a strong ROS-scavenging activity through catalase and peroxidase enzymes, inducing heat shock proteins for cellular homeostasis and, more importantly, by maintaining high levels of PUFA for cell membrane stability and fluidity through a coordinated up-regulation of FAD2 and down-regulation of GDSL-lipase genes. The modulation of the expression levels of these key genes may be orchestrated by transcription factors from the families of NAC, WRKY, HSF and AP2/ERF. A proposed schematic model for the stronger cold response in “Variegatum” is summarized in Figure 8.

## 4. Materials and Methods 

### 4.1. Plant Materials

The naturally occurring variegated cultivar of *Pittosporum tobira* “Variegatum” and the widely grown cultivar “Green Pittosporum” were originally collected from Pingdingshan, Henan in China and used as experimental materials. Biochemical data were recorded on three different plants of each cultivar at different dates corresponding to various temperature gradients (20–10 °C) from August to November (Figure 1). 

### 4.2. Transmission Electron Microscopy (TEM)

TEM analysis was performed as described by Shih et al. [3]. Green and yellow sectors of leaves were cut into small cubes in the field and placed in a fixation solution containing 2.5% glutaraldehyde and 4% paraformaldehyde in 0.1 M sodium phosphate buffer (pH 7.0). Samples underwent 20 min of rinsing three times and were post-fixed in 1% osmium tetroxide for 2 h. After being dehydrated through an ethanol series, samples were infiltrated and embedded in Spurr’s resin and then polymerized at 70 °C for 8 h. Ultrathin sections (~70–90 nm) were collected and stained with ethanol uranyl acetate and lead citrate. The morphology of plastids was observed with Tecnai F20S TEM (The Thermo Scientific™, Waltham, MA, USA) at 200 kV.

### 4.3. Measurement of Physio-Biochemical Parameters

A total of 50 mg fresh leaves were used to extract chlorophyll. The total chlorophyll content (ChlT, mg g**^−^**^1^FW) and carotenoids content (Ca, mg g**^−^**^1^ FW) were determined as described by Wellburn [77]. The net photosynthetic rate (*Pn*, µmol m**^−^**^2^ s**^−^**^1^), intercellular CO_2_ concentration (*Ci*/ppm) and transpiration rate (*Tr*, mmol.m**^−^**^2^ s**^−^**^1^) were determined with a portable L-6400XT (LI-COR, Lincoln, NB, USA). The measurements of photosynthetic parameters were taken at the saturation irradiance with an incident photosynthetic photo flux density (PPFD) of 1200 µmol m**^−^**^2^ s**^−^**^1^ and an airflow rate at 500 µmol s**^−^**^1^. The enzymatic activities of superoxide dismutase (SOD, U g**^−^**^1^), catalase (CAT, U g**^−^**^1^.min**^−^**^1^), peroxidase (POD, U g**^−^**^1^.min**^−^**^1^) and the content of malonaldehyde (MDA, μmol g**^−^**^1^) were calculated by following the manufacturer’s instructions (Biological Engineering Institute of Nanjing Jiancheng, China). Means from three replicates were used for statistical analysis.

### 4.4. RNA Extraction, cDNA Library Construction, and Transcriptome Sequencing

The complete leaves from the cultivars “Green Pittosporum” and “Variegatum” were collected in replicates from three different plants under cold conditions at November 15^th^ (Temperature = 10 °C), immediately frozen in liquid nitrogen and stored at −80 °C until further use. Total RNAs were extracted using Spin Column Plant total RNA Purification Kit following the manufacturer’s protocol (Sangon Biotech, Shanghai, China). Purity of the extracted RNAs was assessed on 1% agarose gels followed by NanoPhotometer spectrophotometer (IMPLEN, Los Angeles, CA, USA). We quantified the RNA using Qubit RNA Assay Kit in Qubit 2.0 Flurometer (Life Technologies, Carlsbad, CA, USA). RNA integrity was checked using the RNA Nano 6000 Assay Kit of the Agilent Bioanalyzer 2100 system (Agilent Technologies, Santa Clara, CA, USA). 

Libraries preparation, and sequencing on Illumina HiSeq 4000 platform (Illumina Inc., San Diego, CA, USA) were performed as described by Zhuang et al. [78].

### 4.5. De novo Assembly, Functional Annotation, Classification and Metabolic Pathway Analysis

Raw transcriptome data were submitted to NCBI SRA, freely accessible at www.ncbi.nlm.nih.gov/bioproject/PRJNA553027. The clean reads were retrieved after trimming adapter sequences, removal of low quality (containing > 50% bases with a Phred quality score < 15) and reads with unknown nucleotides (more than 1% ambiguous residues N) using the FastQC tool (http://www.bioinformatics.babraham.ac.uk/projects/fastqc/). The high-quality reads from all the six libraries were de novo assembled into transcripts using Trinity (Version r20140717) [79] by employing paired-end method. Next, the transcripts were realigned to construct unigenes. The assembled unigenes were then annotated by searching against various databases such as Kyoto Encyclopedia of Genes and Genomes (KEGG) [80], Gene Ontology (GO) [81], Clusters of Orthologous Groups (COG) [82], Pfam [83], Swissprot [84], egNOG [85], NR [86], euKaryotic Orthologous Groups (KOG) [87] using BLAST [88] with a threshold of E-value <1.0 E^−5^.

The software KOBAS2.0 [89] was employed to get the unigene KEGG orthology; the analogs of the unigene amino acid sequences were searched against the Pfam database [83] using HMMER tool [90] with a threshold of E-value < 1.0 E^−10^. The sequenced reads were compared with the unigene library using Bowtie [91], and the level of expression was estimated in combination with RSEM [92]. The gene expression level was determined according to the fragments per kilobase of exon per million fragments mapped (FPKM).

### 4.6. Differential Expression and Enrichment Analysis

The read count was normalized and EdgeR Bioconductor package [93] was used to determine the differential expressed genes (DEGs) between the two cultivars with the fold change of > 2 [94] and false discovery rate correction (FDR) set at *p* < 0.01. GO enrichment analysis was performed using the topGO method [95] based on the wallenius non-central hypergeometric distribution with *p* < 0.05. KEGG pathway enrichment analysis of the DEGs was done using KOBAS2.0 [89]. The FDR correction was employed (*p* < 0.05) to reduce false positive prediction of enriched KEGG pathways.

### 4.7. Validation of Gene Expression Using Quantitative Real Time-PCR

The qRT-PCR was performed on RNA extracted from leaf samples of “Variegatum” and “Green Pittosporum” as described by Dossa et al. [96] using the *Actin* gene as the internal control. Specific primer pairs of ten selected genes were designed using the Primer Premier 5.0 [97] (Table S3). Data are presented as relative transcript level based on the 2^-∆∆*Ct*^ method [98].

### 4.8. Statistical Analysis

Data were analyzed with the R software (www.r-project.org) using the one-way analysis of variance (ANOVA) for significant difference. The error bars were calculated with data from three replicates. ANOVA results were considered significant at *p* < 0.05 and mean comparisons were done using the Tukey HSD test.

## Figures and Tables

**Table 1:** Overview of the transcriptome sequencing dataset and quality check.

Cultivar	Library-ID	Read Number	Base Number	GC (%)	% ≥ Q30
“Green Pittosporum”	T01	22,115,602	6,612,391,012	45.27	92.78
“Green Pittosporum”	T02	23,965,370	7,164,119,518	45.01	92.97
“Green Pittosporum”	T03	22,120,563	6,612,379,716	45.25	92.96
“Variegatum”	T04	23,413,612	6,996,233,542	44.79	93.60
“Variegatum”	T05	22,179,179	6,627,936,224	44.64	93.36
“Variegatum”	T06	22,956,424	6,863,508,574	45.19	92.86

**Table 2:** Statistics of the assembly results.

Length Range	Transcript	Unigene
200–300	53,860(25.64%)	46,279(41.00%)
300–500	38,333(18.25%)	27,916(24.73%)
500–1000	38,190(18.18%)	20,115(17.82%)
1000–2000	39,154(18.64%)	11,929(10.57%)
2000	40,551(19.30%)	6,636(5.88%)
Total number	210,088	112,875
Total length (bp)	241,105,749	72,533,944
N50 length (bp)	2,137	1,017
Mean length (bp)	1147.64	642.60

**Table 3:** Functional annotation statistics of the unigenes.

#Anno_Database	Annotated_Number	300 <= length < 1000	Length >= 1000
COG_Annotation	17,065	6,007	6,317
GO_Annotation	28,283	10,378	9,062
KEGG_Annotation	19,595	7,857	6,277
KOG_Annotation	30,496	11,470	10,018
Pfam_Annotation	33,826	12,283	13,569
Swissprot_Annotation	28,074	10,455	11,043
eggNOG_Annotation	48,410	18,186	15,546
nr_Annotation	47,309	17,751	15,596
All_Annotated	51,718	19,526	15,881

**Table 4:** Statistics of the mapping of sequencing data with assembly results.

Cultivar	Library-ID	Clean Reads	Mapped Reads	Mapped Ratio
“Green Pittosporum”	T01	22,115,602	17,518,471	79.21%
“Green Pittosporum”	T02	23,965,370	19,005,314	79.30%
“Green Pittosporum”	T03	22,120,563	17,599,921	79.56%
“Variegatum”	T04	23,413,612	19,153,207	81.80%
“Variegatum”	T05	22,179,179	17,804,804	80.28%
“Variegatum”	T06	22,956,424	18,290,888	79.68%

**Table 5:** Key DEGs related to the enriched KEGG pathways involved in the cold responses in variegated *P. tobira.*

Pathway	KO	Gene ID	Log2 Fold Change	Gene Description
Phenylalanine metabolism				
	K00815	*c55523.graph_c0*	1.565	Aminotransferase TAT2
	K00430	*c68309.graph_c0*	1.057	Peroxidase
	K00430	*c74970.graph_c1*	1.157	Peroxidase
Sesquiterpenoid and triterpenoid biosynthesis				
	K15472	*c29794.graph_c0*	1.488	Premnaspirodiene oxygenase, Cytochrome P450
	K15472	*c43399.graph_c0*	1.198	Premnaspirodiene oxygenase, Cytochrome P450
Biosynthesis of unsaturated fatty acids and fatty acid metabolism				
	K10256	*c45880.graph_c0*	1.973	FAD2
	K10256	*c72696.graph_c0*	1.287	FAD2
	K10256	*c74068.graph_c0*	1.493	FAD2
	K10256	*c74296.graph_c0*	1.385	FAD2
	K10256	*c75682.graph_c0*	1.284	FAD2
	K10256	*c45880.graph_c0*	1.973	FAD2
	K10256	*c72696.graph_c0*	1.287	FAD2
	K10256	*c74068.graph_c0*	1.493	FAD2
	K10256	*c74296.graph_c0*	1.385	FAD2
	K10256	*c75682.graph_c0*	1.284	FAD2
	−−	*c52980.graph_c1*	−1.179	GDSL
	−−	*c52915.graph_c0*	−1.582	GDSL
	−−	*c62735.graph_c0*	−2.134	GDSL
	−−	*c28930.graph_c0*	−1.998	GDSL
	−−	*c79198.graph_c0*	−1.068	GDSL
	−−	*c63752.graph_c0*	−1.502	GDSL
	−−	*c28455.graph_c0*	−1.267	GDSL
	−−	*c71358.graph_c0*	−1.635	GDSL
	−−	*c76303.graph_c0*	−1.713	GDSL
Protein processing in endoplasmic reticulum				
	K13993	*c55937.graph_c0*	−2.839	22.7kDa HSP IV
	K13993	*c60091.graph_c0*	−1.125	15 kDa HSP
	K13993	*c60491.graph_c0*	−1.961	17.9 kDa HSP II
	K13993	*c70233.graph_c1*	−1.292	18.1 kDa HSP I
	K04079	*c74041.graph_c0*	1.283	90 kDa HSP
	K03283	*c75081.graph_c0*	1.095	70 kDa HSP
	K09489	*c68688.graph_c0*	1.266	70 kDa HSP
	K03283	*c28616.graph_c1*	1.018	70 kDa HSP
	K03283	*c69810.graph_c6*	1.408	70 kDa HSP

## Supplementary Materials

Supplementary Materials can be found at https://www.mdpi.com/1422-0067/20/19/4857/s1. Table S1. Full list of the unigenes annotated in *Pittosporum tobira* leaf; Table S2. Full list of the genes expressed in *Pittosporum tobira* leaf and their FPKM values. T01-T03 represent the three replicates libraries of the cultivar “Green Pittosporum” and T04-T06 represent the three replicates libraries of the cultivar “Variegatum”; Table S3. The primer sequences of genes used for real time quantitative PCR; Figure S1. qRT-PCR results of 10 selected genes and correlation between transcriptome data and real time PCR results.

## Abbreviations

CAT	Catalase
DEG	Differentially expressed gene
FAD2	Fatty acid desaturase 2
FPKM	Fragments per kilobase of exon per million fragments mapped
GO	Gene ontology
HSF	Heat shock factor
HSP	Heat shock protein
KEGG	Kyoto Encyclopedia of Genes and Genomes
MDA	Malonaldehyde
POD	Peroxidase
PUFA	Poly-unsaturated fatty acids
ROS	Reactive oxygen species
SOD	Superoxide dismutase
TF	Transcription factor
