# Supplementary figures and images for: Protective Role of Leaf Variegation in Pittosporum tobira under Low Temperature: Insights into the Physio-Biochemical and Molecular Mechanisms
Source: Int J Mol Sci. 2019 Sep 30;20(19):4857. doi: 10.3390/ijms20194857 (PMC6801658; doi:10.3390/ijms20194857)

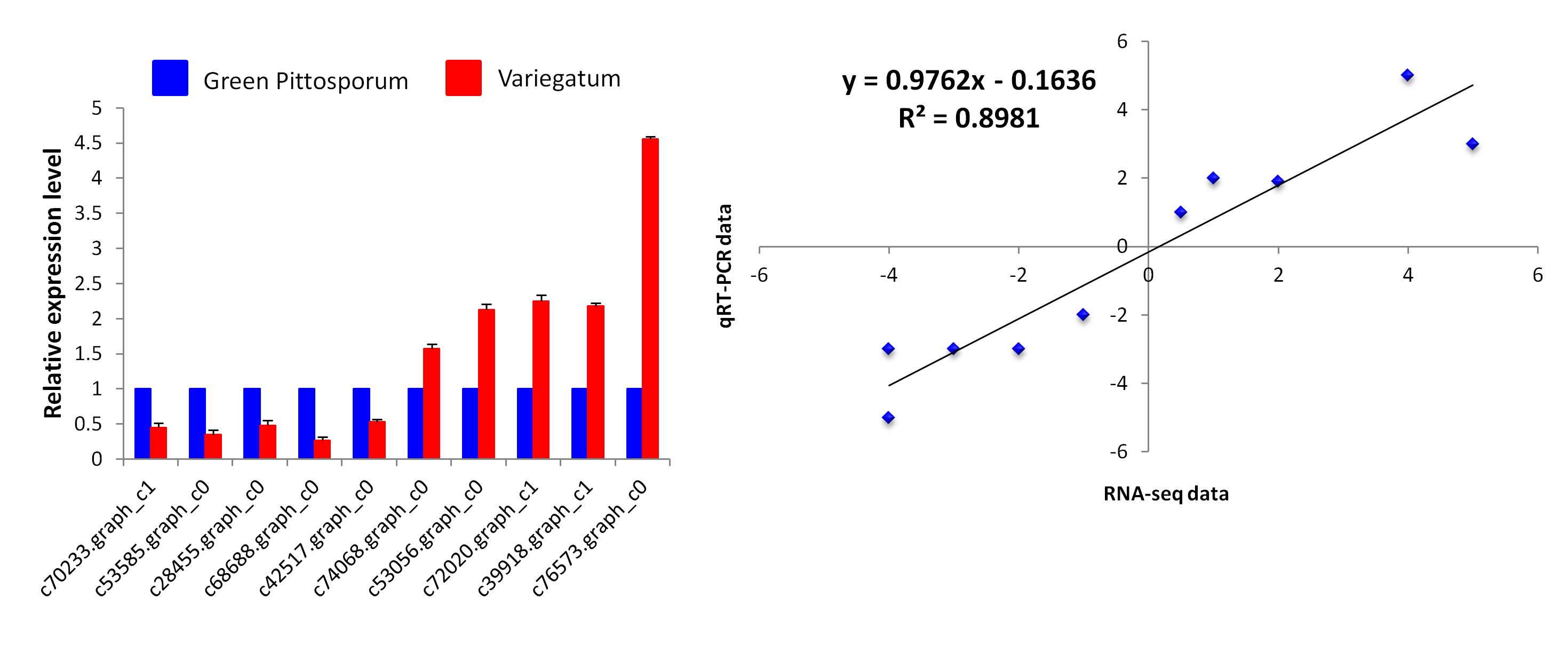

Supplement: Supplementary file 1 [file ijms-20-04857-s001.zip › Figure S1.tif]
